# Leveraging Retrieval-Augmented Large Language Models for Dietary Recommendations With Traditional Chinese Medicine’s Medicine Food Homology: Algorithm Development and Validation

**DOI:** 10.2196/75279

**Published:** 2025-08-21

**Authors:** Hangyu Sha, Fan Gong, Bo Liu, Runfeng Liu, Haofen Wang, Tianxing Wu

**Affiliations:** 1 School of Computer Science and Engineering, Southeast University, 2 Southeast University Road, Jiangning District, Nanjing, 210096, China, 86 15077889931; 2 Key Laboratory of New Generation Artificial Intelligence Technology and Its Interdisciplinary Applications (Southeast University), Ministry of Education, China; 3 Department of Endocrinology, Shuguang Hospital Affiliated to Shanghai University of Traditional Chinese Medicine, Shanghai, China; 4 Informatization Office, Shanghai University of Traditional Chinese Medicine, Shanghai, China; 5 College of Design and Innovation, Tongji University, Shanghai, China

**Keywords:** Traditional Chinese Medicine, medicine food homology, large language model, retrieval-augmented generation, uncertain knowledge graph, dietary recommendation

## Abstract

**Background:**

Traditional Chinese Medicine (TCM) emphasizes the concept of medicine food homology (MFH), which integrates dietary therapy into health care. However, the practical application of MFH principles relies heavily on expert knowledge and manual interpretation, posing challenges for automating MFH-based dietary recommendations. Although large language models (LLMs) have shown potential in health care decision support, their performance in specialized domains such as TCM is often hindered by hallucinations and a lack of domain knowledge. The integration of uncertain knowledge graphs (UKGs) with LLMs via retrieval-augmented generation (RAG) offers a promising solution to overcome these limitations by enabling a structured and faithful representation of MFH principles while enhancing LLMs’ ability to understand the inherent uncertainty and heterogeneity of TCM knowledge. Consequently, it holds potential to improve the reliability and accuracy of MFH-based dietary recommendations generated by LLMs.

**Objective:**

This study aimed to introduce Yaoshi-RAG, a framework that leverages UKGs to enhance LLMs' capabilities in generating accurate and personalized MFH-based dietary recommendations.

**Methods:**

The proposed framework began by constructing a comprehensive MFH knowledge graph (KG) through LLM-driven open information extraction, which extracted structured knowledge from multiple sources. To address the incompleteness and uncertainty within the MFH KG, UKG reasoning was used to measure the confidence of existing triples and to complete missing triples. When processing user queries, query entities were identified and linked to the MFH KG, enabling retrieval of relevant reasoning paths. These reasoning paths were then ranked based on triple confidence scores and entity importance. Finally, the most informative reasoning paths were encoded into prompts using prompt engineering, enabling the LLM to generate personalized dietary recommendations that aligned with both individual health needs and MFH principles. The effectiveness of Yaoshi-RAG was evaluated through both automated metrics and human evaluation.

**Results:**

The constructed MFH KG comprised 24,984 entities, 22 relations, and 29,292 triples. Extensive experiments demonstrate the superiority of Yaoshi-RAG in different evaluation metrics. Integrating the MFH KG significantly improved the performance of LLMs, yielding an average increase of 14.5% in Hits@1 and 8.7% in *F*_1_-score, respectively. Among the evaluated LLMs, DeepSeek-R1 achieved the best performance, with 84.2% in Hits@1 and 71.5% in *F*_1_-score, respectively. Human evaluation further validated these results, confirming that Yaoshi-RAG consistently outperformed baseline models across all assessed quality dimensions.

**Conclusions:**

This study shows Yaoshi-RAG, a new framework that enhances LLMs’ capabilities in generating MFH-based dietary recommendations through the knowledge retrieved from a UKG. By constructing a comprehensive TCM knowledge representation, our framework effectively extracts and uses MFH principles. Experimental results demonstrate the effectiveness of our framework in synthesizing traditional wisdom with advanced language models, facilitating personalized dietary recommendations that address individual health conditions while providing evidence-based explanations.

## Introduction

### Background

The concept of “medicine food homology” (MFH) in Traditional Chinese Medicine (TCM) means that certain substances can function as both nutritional food and therapeutic medicines depending on their application and dosage [1]. This ancient philosophy has gained considerable traction in modern health care systems [2], offering approaches to disease prevention and management through dietary interventions [3]. However, the practical implementation of MFH principles remains largely dependent on the expert knowledge of TCM practitioners, leading to inconsistent application and limited accessibility. The absence of automated and systematic approaches to using MFH principles for dietary recommendation generation has hindered its broader integration into contemporary health care frameworks and constrained its potential benefits for public health.

Recent advances in large language models (LLMs) [4] have demonstrated their remarkable performance across a variety of medical tasks [5], including automated clinical decision support [6], medical education [7], and health care information retrieval [8]. These capabilities are primarily derived from extensive pretraining on large-scale corpora, which encode rich knowledge from diverse textual sources. The application of MFH principles heavily depends on the domain knowledge documented in TCM literature, including foods, herbs, and formulations. Given that many LLMs have been pretrained on the corpora, which incorporate such TCM literature, combined with their strong natural language generation abilities, they present promising potential for automating MFH-based dietary recommendations. Nonetheless, LLMs exhibit substantial limitations as they often produce hallucinations that refer to factually incorrect information [9]. This problem is especially evident when generating information related to the medicinal properties of food in the context of TCM. For example, when querying with “I have stomach discomfort recently, can you recommend some summer drinks?” the LLMs respond: “Mung bean soup clears heat and is suitable for heat-related discomfort.” However, according to MFH principles, mung beans are cold (yin), and their consumption may aggravate stomach discomfort. Such inaccuracies significantly affect the safety and reliability of LLMs in practical health care applications [10].

Retrieval-augmented generation (RAG) has emerged as a promising strategy to mitigate these limitations by incorporating external knowledge into LLMs, thereby enhancing factual accuracy and reducing hallucinations [11]. However, using TCM knowledge as the retrieval source of RAG presents unique challenges. TCM knowledge is sourced from diverse and heterogeneous materials, including ancient and classical literature, modern TCM literature, food and herbs information, regulatory documents, research papers, and TCM formulations. This multisource nature results in inconsistent knowledge representation, terminological ambiguities, and varying levels of evidence quality. Furthermore, MFH principles inherently involve uncertainties arising from dosage effects, individual variability, and context-dependent applications [12]. Currently, there is no comprehensive and structured knowledge base specifically tailored for supporting RAG grounded in MFH principles, creating a significant gap between traditional wisdom and modern technologies.

### Related Work

#### Dietary Recommendation Systems

Dietary recommendation systems provide personalized food and nutrition suggestions based on individual health status and preferences. Traditional approaches have primarily relied on rule-based methods using expert knowledge and predefined guidelines, often lacking personalization and accessibility. Recent advancements in artificial intelligence have transformed this field through deep learning and knowledge graphs (KGs). Chen et al [13] defined food recommendation as constrained question answering over a food KG, integrating user preferences with health guidelines. PPKG [14] is a cancer-specific dietary recommendation system that combines a KG with time-aware long short-term memory networks to dynamically adjust recommendations for cancer prevention and rehabilitation. Ma et al [15] proposed a nutrition-related KG method based on graph convolutional networks to enhance food recommendation diversity while promoting more healthy eating habits. LLMs have further advanced dietary recommendation capabilities. ChatDiet [16] integrates personal and population models with causal inference to enhance personalization and explainability. Jin et al [17] demonstrated the clinical effectiveness of a GPT-based dietary recommendation system for patients with hemodialysis, successfully reducing serum potassium levels. Kopitar et al [18] created a generative artificial intelligence system for personalized inpatient meal planning that incorporates electronic health records and clinical guidelines.

However, we still do not have methods to integrate MFH principles with LLMs for dietary recommendation. Our framework bridges this gap by combining MFH KG with LLMs to develop more comprehensive and culturally informed dietary recommendations.

#### The Interplay Between LLMs and KGs

KGs serve as structured representations of factual knowledge, providing an excellent complement to parameterized language models [19,20]. With the emergence of LLMs, research has evolved along 2 complementary trajectories: leveraging LLMs to construct KGs and enhancing LLMs with KG integration. Several approaches have emerged for LLM-assisted KG construction. Extract-define-canonicalize [21] addresses scalability through open information extraction (OpenIE) and post hoc canonicalization. BEAR [22] leverages LLMs for zero-shot knowledge extraction in service-oriented domains. LLM-TIKG [23] applies few-shot learning for constructing threat intelligence KGs, while Yang et al [24] presented an approach for medical ontology expansion using LLMs to generate competency questions.

Researchers have also explored integrating KGs to enhance LLMs’ capabilities. GraphGPT [25] combines LLMs with graph structural knowledge through instruction tuning. GaLM [26] transforms KGs into text representations to improve reasoning while reducing hallucinations. Think-on-Graph [27] is a training-free framework that uses iterative beam search to enable interactive reasoning, while StructGPT [28] enhances zero-shot reasoning through an iterative Reading-then-Reasoning approach. Our work builds upon these dual research trajectories by proposing a framework that leverages LLMs for KG construction and uses KGs to enhance the capabilities of LLMs specifically within the domain of TCM, thereby providing reliable dietary recommendations grounded in MFH principles.

### Objectives

To overcome the limitations of existing methods, we propose a new framework that enhances LLMs’ capability to generate MFH dietary recommendations by using an uncertain knowledge graph (UKG) as an external resource. This framework uses multistep LLM calls for OpenIE [29] to automatically construct an MFH KG from unstructured and heterogeneous literature sources. It then implements UKG reasoning to measure the confidence of existing triples and complete missing triples. To improve knowledge retrieval, the framework adopts a reasoning path–based knowledge retrieval strategy, extracting relevant knowledge from the MFH KG and refining the results through ranking and filtering based on triple confidence scores and entity importance. By encoding domain-specific knowledge and addressing uncertainties related to MFH, the MFH KG empowers LLMs to deliver more personalized, evidence-based, and reliable dietary recommendations.

The key contributions of this study are as follows: (1) an integrated framework that leverages KG-augmented LLMs to generate personalized and evidence-based MFH dietary recommendations, (2) an LLM-driven OpenIE methodology for automatically constructing a UKG specifically tailored to TCM’s MFH from heterogeneous multisource data, (3) a systematic evaluation of several mainstream LLMs for the generation of MFH dietary recommendations, and (4) experimental results and case studies that demonstrate the capability of the proposed framework to deliver personalized and professional MFH-based dietary recommendations.

## Methods

### Overview

**Figure 1.**
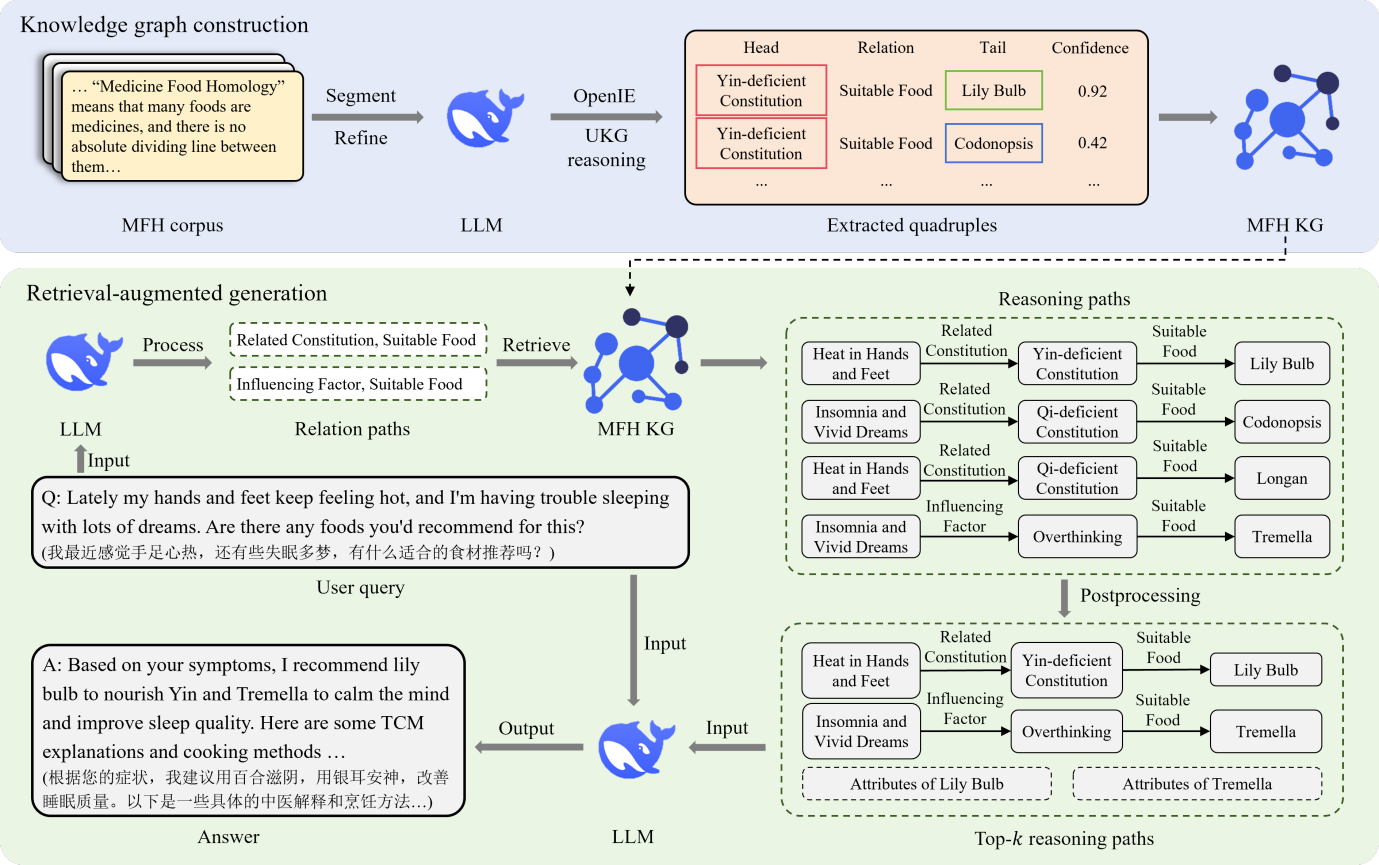
The architecture of the proposed framework. KG: knowledge graph; LLM: large language model; MFH: medicine food homology; OpenIE: open information extraction; TCM: Traditional Chinese Medicine; UKG: uncertain knowledge graph.

This study proposed Yaoshi-RAG, an RAG framework designed to enhance LLMs’ capabilities in generating evidence-based dietary recommendations based on MFH principles. As illustrated in Figure 1, the framework consists of 2 modules:

1. Knowledge graph construction: A comprehensive corpus was compiled from multiple sources, and an MFH KG was constructed using LLM-driven OpenIE. Then, UKG reasoning was applied to measure the confidence of extracted triples and complete missing triples.
2. Retrieval-augmented generation: Given a user query, the framework first identified and linked query entities to the MFH KG. The LLM then constructed relation paths to facilitate the retrieval of reasoning paths. Subsequently, these candidate reasoning paths were refined through postprocessing, including ranking and filtering, to extract the top-k most relevant reasoning paths, thereby improving retrieval accuracy. The retrieved knowledge was integrated with the user query through prompt engineering, guiding the LLM to generate personalized dietary recommendations that aligned with both the user’s needs and MFH principles.

A comprehensive corpus was compiled from multiple sources, and an MFH KG was constructed using LLM-driven OpenIE. Then, UKG reasoning was applied to measure the confidence of extracted triples and complete missing triples. In the following sections, we provide a detailed explanation of both modules, outlining their design, implementation, and contributions to the overall framework.

### KG Construction

#### Corpus Collection

To construct an MFH KG, we systematically constructed a corpus from diverse sources, including ancient and classical literature, modern TCM literature, food and herbs information, regulatory documents, research papers, and TCM formulations. This process results in a corpus of 1359 relevant documents. Table 1 shows the statistical distribution of documents across different source categories.

**Table 1.** The statistical distribution of 1359 documents.

Source category	Number of documents
Ancient and classical literature	75
Modern TCM^a^ literature	228
Food and herbs information	400
Regulatory documents	42
Research papers	432
TCM formulations	182
Total	1359

a TCM: Traditional Chinese Medicine.

To improve the efficiency and accuracy of entity-relation triple extraction, each document was segmented into passages of 300 tokens. These passages were then input into LLMs with a carefully designed prompt (Textbox S1 in Multimedia Appendix 1) to resolve pronominal references by incorporating information from document titles and neighboring passages. Additionally, irrelevant content within passages was filtered out to ensure domain-specific focus.

#### MFH Knowledge Graph

**Figure 2.**
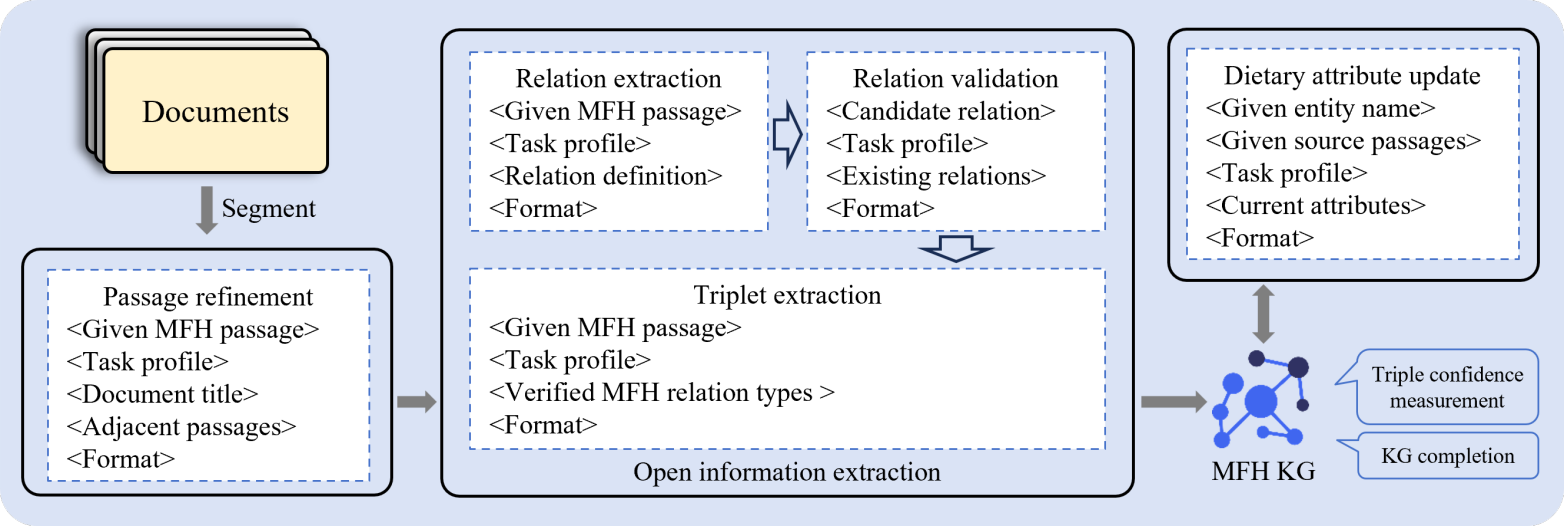
The detailed process of medicine food homology knowledge graph construction. KG: knowledge graph; MFH: medicine food homology.

Based on the collected corpus, we constructed an MFH KG G, as shown in Figure 2, which illustrates the detailed process. Formally, the graph is defined as a triple G=(E,R,F), where E represents the set of entities, R represents the set of relations, and F⊆E×R×E represents the fact set. Each fact in F is represented as a quadruple (h,r,t,s), where h is the head entity, r is the relation, t is the tail entity, and s is the confidence score reflecting the likelihood that the triple (h,r,t) holds true. For instance, consider the quadruple (h: Insomnia and Vivid Dreams, r: Related Constitution, t: Yin-Deficient Constitution, s: 0.87). This quadruple indicates that Insomnia and Vivid Dreams are associated with the Yin-Deficient Constitution, with a confidence score of 0.87.

We used LLM-driven OpenIE to extract triples $\left(h,r,t\right)$ from each passage within the corpus. Specifically, we first integrated predefined relation definitions into a prompt (Textbox S2 in Multimedia Appendix 1), guiding the LLM to identify and extract potential relations. These candidate relations, along with existing ones, were subsequently processed using another prompt (Textbox S3 in Multimedia Appendix 1), where the LLM determined whether to retain new relations or filter out redundant ones. This process resulted in the construction of a relation set $r_{sel}$, which was incorporated into OpenIE prompts, instructing the LLM to extract entity-relation triples. The relations of extracted triples were strictly constrained to the relation set $r_{sel}$.

Specifically, during the OpenIE, we instructed the LLM to extract attributes of food entities and dish entities (Textboxes S4 and S5 in Multimedia Appendix 1). For food entities, LLMs referenced their source passages to update attributes, including name, primary effects, consumption methods, indications, and contraindications. Similarly, for dish entities, attributes such as name, ingredient composition, cooking method, and dietary benefits were updated.

The automated construction of the MFH KG inevitably introduced noise, incompleteness, and factual inaccuracies, making absolute factual correctness unattainable. To mitigate these issues, we adopted UKG reasoning to measure the confidence of extracted triples and complete missing triples. Specifically, we first used unKR [30], an open-source Python library for UKG reasoning, to learn vector representations of entities and relations. Based on these representations, we applied the triple confidence measurement method [31], integrating heterogeneous evidences to measure confidence of each triple. For each given head entity h and relation r, candidate tail entities were enumerated to construct new triples absent from the current graph. The triples with confidence scores larger than 0.85 were added to the MFH KG.

### Retrieval-Augmented Generation

#### The Retrieval Strategy

Given a user query Q, we first constructed a prompt (Textbox 1) to guide the LLM to extract a set of relevant keywords E from Q. We then encoded both the keywords and all entities from the MFH KG into dense vector representations, denoted as $H_{R}$ and $H_{G}$, respectively. Considering that SBERT [32] has demonstrated superior performance over BERT [33] and RoBERTa [34] on semantic textual similarity tasks while maintaining lower computational overhead [35], we adopted SBERT for embedding both the extracted keywords and entities. Subsequently, we computed the cosine similarities between the embeddings of each keyword and all entities. For each keyword, we retrieved the top-10 most similar entities from the MFH KG based on cosine similarity scores. These candidate entities were then presented to the LLM, which was instructed to identify the matched entity (Textbox 2). The selected entity corresponding to each keyword constitutes the query entity set $E_{Q}=\left\{e_{1},e_{2},\ldots \;,e_{n}\right\}$, which serves as the starting point for subsequent reasoning path retrieval.

Textbox 1. **Prompt for keyword extraction.**

You are a medical knowledge assistant specialized in Traditional Chinese Medicine. Given a user query, extract a concise set of keywords that represent the user's core health-related intent and information needs. These keywords will be used to retrieve relevant knowledge from a medicine-food homology knowledge graph to answer the user query.

User query: {user_query}

Please return the output as a Python-style set of keywords:

Textbox 2. **Prompt for entity linking.**

Given a keyword and a list of candidate entities retrieved from the medicine food homology knowledge graph, select the entity that most accurately matches the keyword based on contextual meaning and traditional usage. If none of the entities are appropriate matches, return "None."

Keyword: {keyword}

Candidate entities: {candidate_entities}

Please return the output as a JSON list:

This study used path-based retrieval from the MFH KG rather than retrieving contextual subgraphs for linked entities. This is motivated by the advantages reasoning paths offer in generating MFH-based dietary recommendations. Specifically, reasoning paths provide structured knowledge by capturing direct relations between query entities and answer entities. For example, the reasoning path “(Insomnia and Vivid Dreams, Related Constitution, Qi-deficient Constitution); (Qi-deficient Constitution, Suitable Food, Codonopsis)*”* enables LLMs to clearly infer the relations between a user’s symptoms, their constitution, and the recommended food, thereby enhancing the interpretability. In contrast, subgraph-based retrieval in long-hop reasoning tasks often introduces irrelevant information [36]. This not only leads to significant computational overhead but also hinders LLMs from effectively extracting relevant information from the retrieval results.

Built upon the previous work [37], we first provided the LLM with a structured prompt (Textbox 3) listing all relations in G. The LLM then generated multiple relation paths $\;\left\{p_{rel}^{1},\;p_{rel}^{2},\;\ldots ,\;p_{rel}^{k}\right\}$, where each $p_{rel}^{i}$ is an ordered sequence of relations$\left\{r_{1},r_{2},\ldots ,r_{l}\right\}$. These relation paths serve as guides for retrieving knowledge essential to answering user queries. Given that the MFH KG contained considerably fewer relations compared with large-scale KGs such as FreeBase [38], we did not fine-tune the LLM for relation path generation. Instead, we used few-shot prompting to enhance the LLM’s ability to generate meaningful relation paths.

Textbox 3. **Prompt for relation paths generation.**

Task instructions:

1. Please generate multiple relation paths (sequences of relations) based on the existing relation types. Each relation path should contain relations that are connected through the same entity.
2. The purpose of the relation paths is to assist in retrieving relevant entities that can answer the user query.

Relation types: {existing_relations}

User query: {user_query}

Query entities: {query_entities}

Few-shot examples: {examples}

Please strictly follow the task instructions and return only the required JSON list:

Subsequently, starting from each entity in the $E_{Q}$, we followed each generated relation path $p_{rel}^{i}$ to retrieve a set of reasoning paths $P_{rea}=\left\{p_{rea}^{1},\;p_{rea}^{2},\;\ldots ,\;p_{rea}^{m}\right\}$ from G, where each reasoning path $p_{rea}^{i}=\left\{\left(e_{1},r_{1},e_{2}\right),\left(e_{2},r_{2},e_{3}\right),\ldots ,\left(e_{l},r_{l},e_{l+1}\right)\right\}$ consists of multiple triples $\left(e_{j},r_{j},e_{j+1}\right)$ and represents an instance of one of the relation paths.

#### Retrieval Result Postprocessing

In our initial experiment, we noticed that the reasoning paths in $P_{rea}$ contained redundant and irrelevant information. To enhance the quality of these reasoning paths, we computed a score for each reasoning path in $P_{rea}$ by considering both the entity importance and the triple confidence within the reasoning path. These scores enabled us to filter out less relevant reasoning paths.

The importance of each entity in the reasoning path was calculated using a weighted PageRank [39] algorithm. Traditional PageRank ignored the confidence of triples, which could lead to equivalent treatment of false and true relations, thus causing inaccuracies in entity importance. To overcome this limitation, we constructed a subgraph using entities from the $P_{rea}$ along with their one-hop neighbors and incorporated confidence scores of triples as edge weights in the PageRank computation. The weighted PageRank formula is defined as:


(1) $$\begin{array}{c} PR\left(e_{i}\right)=\left(1-d\right)\cdot \frac{1}{N}+d\cdot \sum_{e_{j}\subset In\left(e_{i}\right)}\frac{w_{j\to i}}{\sum_{k}w_{j\to k}}\cdot PR\left(e_{j}\right) \end{array}$$

where $PR\left(e_{i}\right)$ represents the weighted PageRank score of entity $e_{i}$, d is the damping factor, and N is the total number of entities in the graph. The term $w_{j\to i}$ denotes the edge weight from entity $e_{i}$ to $e_{j}$, derived from the confidence score of the corresponding triples. When multiple relations exist between $e_{i}$ and $e_{j}$ with different confidence scores, their influence was aggregated by averaging these scores. For scoring the reasoning paths, we integrated entity importance and triple confidence scores to formulate the following path-scoring function:


(2) $$\begin{array}{c} PathScore\left(P\right)=\prod_{i=1}^{n}w_{i-1\to i}\times \left(\frac{1}{n}\sum_{e_{j}\in P}PR\left(e_{j}\right)\right) \end{array}$$

where $w_{i-1\to i}$ represents the confidence score of the relation from entity $e_{i}$ to $e_{j}$, n denotes the total number of entities in the path, and $PR\left(e_{j}\right)$ is the weighted PageRank score of entity $e_{j}$ within the reasoning path. This scoring methodology captured both the confidence of triples within the reasoning path and the importance of the entities involved, striking a balance between path reliability and informational importance. Finally, we ranked the reasoning paths based on their scores and retained only the top-k reasoning paths for subsequent inference.

#### Answer Generation

We used prompt engineering to improve the accuracy and relevance of generated answers of LLM. Prompt engineering involves constructing carefully designed prompts to guide LLMs toward generating high-quality and task-specific responses. Specifically, we constructed a prompt template (Textbox 4) comprising four key components: (1) task specification, (2) user query, (3) retrieved reasoning paths, and (4) attributes of food and dish entities. This structured prompt facilitates the integration of external knowledge, improves the LLMs’ comprehension of MFH principles, and supports the generation of more accurate responses.

Textbox 4. **Prompt for answer generation.**

You are a professional Traditional Chinese Medicine doctor, providing dietary recommendations using the concept of “medicine food homology.” Given a user query and the relevant reasoning paths and entity attributes, generate an answer.

Task instructions:

1. Please provide a detailed, accurate, and concise response based on the information provided.
2. The response should be well-structured and ensure logical consistency with the given reasoning paths and entity attributes.
3. Incorporate medicine food homology principles in your recommendations.
4. Output format: {“answer”: “(Your answer here.)”}. The answer must be written in Chinese.

User query: {user_query}

Reasoning paths: {reasoning_paths}

Entity attributes: {entity_attributes}

Please strictly follow the task instructions and return only the required JSON format:

### LLM Selection

To identify the most suitable LLM for MFH dietary recommendation tasks, we evaluated several mainstream LLMs, including GPT-4 [40], LLaMA2-Chat-7B [41], Qwen2.5-7B [42], and DeepSeek-R1 [43]. Additionally, we considered the influence of model size, incorporating both large-scale models (GPT-4 and DeepSeek-R1) and 7B-parameter models (LLaMA2-Chat-7B and Qwen2.5-7B). During the experiment, we implemented the same prompt set across all models to ensure comparative evaluation under identical input conditions. All models were integrated with our proposed framework for dietary recommendation generation based on the MFH principles.

### Evaluation Metrics

#### Automated Evaluation

To quantitatively assess the effectiveness and accuracy of the model’s dietary recommendations, we used 2 evaluation metrics: Hits@1 and *F*_1_-score. Hits@1 measures the proportion of queries where the top-ranked predicted recommendation matches a ground truth answer. This metric evaluates the model’s ability to prioritize the most relevant dietary recommendation. *F*_1_-score captures the harmonic mean of precision (P) and recall (R), offering a balanced evaluation of both aspects. This metric is particularly appropriate in scenarios where multiple valid recommendations may exist for a single query. It is defined as follows:


(3) $$\begin{array}{c} F_{1}=2\times \frac{P\times R}{P+R} \end{array}$$

where precision and recall are defined as:


(4) $$\begin{array}{c} P=\frac{TP}{TP+FP},\;R=\frac{TP}{TP+FN} \end{array}$$

In this context, *TP* (true positives) represents the number of dietary recommendations correctly suggested, FP (false positives) indicates inappropriate dietary recommendations incorrectly suggested, and FN (false negatives) refers to appropriate dietary recommendations that the model failed to suggest.

#### Human Evaluation

While automated evaluation methods are widely applied in natural language processing tasks, they have certain limitations in the context of dietary recommendations. Automated metrics struggle to assess the effectiveness of recommendations, specifically whether the suggested food and their combinations align with MFH principles. Moreover, user acceptability and explainability of the generated dietary recommendations are difficult to accurately assess using automated methods. Therefore, we incorporated human evaluation, integrating subjective ratings from both experts and general users to provide a more comprehensive assessment of the generated dietary recommendations.

Specifically, we assessed the generated dietary plans based on the following five evaluation criteria: (1) rationality, (2) explainability, (3) user acceptability, (4) personalization, and (5) consistency. Rationality assesses whether the recommended dietary plan adhered to both TCM principles and modern nutritional standards. Explainability evaluates whether the recommendations included clear TCM-based justifications. A higher score indicates that the system provided reasonable and understandable explanations rather than simply outputting ingredient combinations. User acceptability measures how well the recommendations aligned with users’ subjective preferences, including taste, cultural adaptability, and feasibility. Personalization assesses whether the system provided tailored recommendations based on an individual’s constitution and health conditions. Consistency examines whether the model produced stable recommendations under similar inputs.

Among these evaluation criteria, rationality, explainability, user acceptability, and personalization were assessed using a 5-point Likert scale (1‐5, where 1 represented the lowest score and 5 represented the highest). Consistency was evaluated using a binary judgment (Yes=5 points and No=0 points).

To conduct the evaluation, we recruited 5 experts with backgrounds in TCM or nutrition, along with 20 general users. Participants followed a structured human evaluation guideline to rate the generated dietary recommendations. The evaluation was conducted using a survey system, where assessors were provided with the user queries along with the corresponding dietary recommendations generated by LLMs. Finally, we computed the mean scores for each evaluation criterion.

### Ethical Considerations

Institutional review board approval was not required for this study, as it did not involve direct research with human participants. All corpora used, such as research papers and TCM formulations, were publicly available [44-46], and no identifiable private or sensitive information was accessed or recorded. Annotators involved in the human evaluation participated voluntarily, provided informed consent prior to participation, and were informed of their right to withdraw at any time. No compensation was provided, and no private information was collected.

## Results

We constructed an MFH KG comprising 24,984 entities, 22 relations, and 29,292 triples. To evaluate the LLMs’ performance in dietary recommendation tasks, we developed a specialized question answering dataset consisting of 2000 queries focused on MFH principles. This dataset was designed to ensure balanced coverage across diverse health conditions and TCM constitutional types.

Table 2 shows a comparative analysis of LLMs with or with no augmentation by the MFH KG on dietary recommendation tasks. Overall, the integration of the MFH KG substantially improved model performance across all evaluated metrics, with an average increase of 14.5% in Hits@1 and 8.7% in *F*_1_-score, respectively. Among the baseline LLMs, DeepSeek-R1 achieved the best performance (Hits@1: 70.4%, *F*_1_-score: 64.1%), followed by GPT-4 (Hits@1: 68.6%, *F*_1_-score: 55.8%), Qwen2.5-7B (Hits@1: 58.7%, *F*_1_-score: 53.2%), and LLaMA2-Chat-7B (Hits@1: 54.1%, *F*_1_-score: 49.3%). The performance ranking remained consistent after MFH KG augmentation, with DeepSeek-R1 outperforming the others (Hits@1: 84.2%, *F*_1_-score: 71.5%). Due to its superior performance in the above automated evaluation, DeepSeek-R1 was selected as the LLM for all subsequent experiments.

**Table 2.** The performance comparison between large language models and medicine food homology knowledge graph–augmented large language models on dietary recommendation tasks.

Model	Hits@1	*F*_1_-score
LLMs^a^		
GPT-4	68.6	55.8
LLaMA2-Chat-7B	54.1	49.3
Qwen2.5-7B	58.7	53.2
DeepSeek-R1	70.4	64.1
MFH^b^ KG^c^+ LLMs		
GPT-4	82.6	69.4
LLaMA2-Chat-7B	70.3	55.6
Qwen2.5-7B	72.8	60.6
DeepSeek-R1	*84.2* ^*d*^	*71.5*

a LLM: large language model.

b MFH: medicine food homology.

c KG: knowledge graph.

d Values in italics indicate the best performance for each metric.

In human evaluation, we conducted a comparative assessment between LLMs and MFH KG–augmented LLMs across 5 key metrics. As shown in Table 3, the MFH KG–augmented DeepSeek-R1 consistently outperformed the baseline DeepSeek-R1 model across all evaluation criteria. The MFH KG–augmented model achieved substantially higher scores in rationality (4.1 vs 3.2), explainability (4.3 vs 2.9), user acceptability (4.4 vs 3.6), personalization (4.2 vs 3.3), and consistency (4.6 vs 3.4). These results provide compelling evidence that augmenting DeepSeek-R1 with the specialized MFH KG significantly improves the quality of dietary recommendations based on MFH principles.

**Table 3.** The performance comparison between DeepSeek-R1 and medicine food homology knowledge graph–augmented DeepSeek-R1 by human evaluation.

Models	Rationality	Explainability	User acceptability	Personalization	Consistency
DeepSeek-R1	3.2	2.9	3.6	3.3	3.4
MFH^a^ KG^b^ + DeepSeek−R1	4.1	4.3	4.4	4.2	4.6

a MFH: medicine food homology.

b KG: knowledge graph.

**Figure 3.**
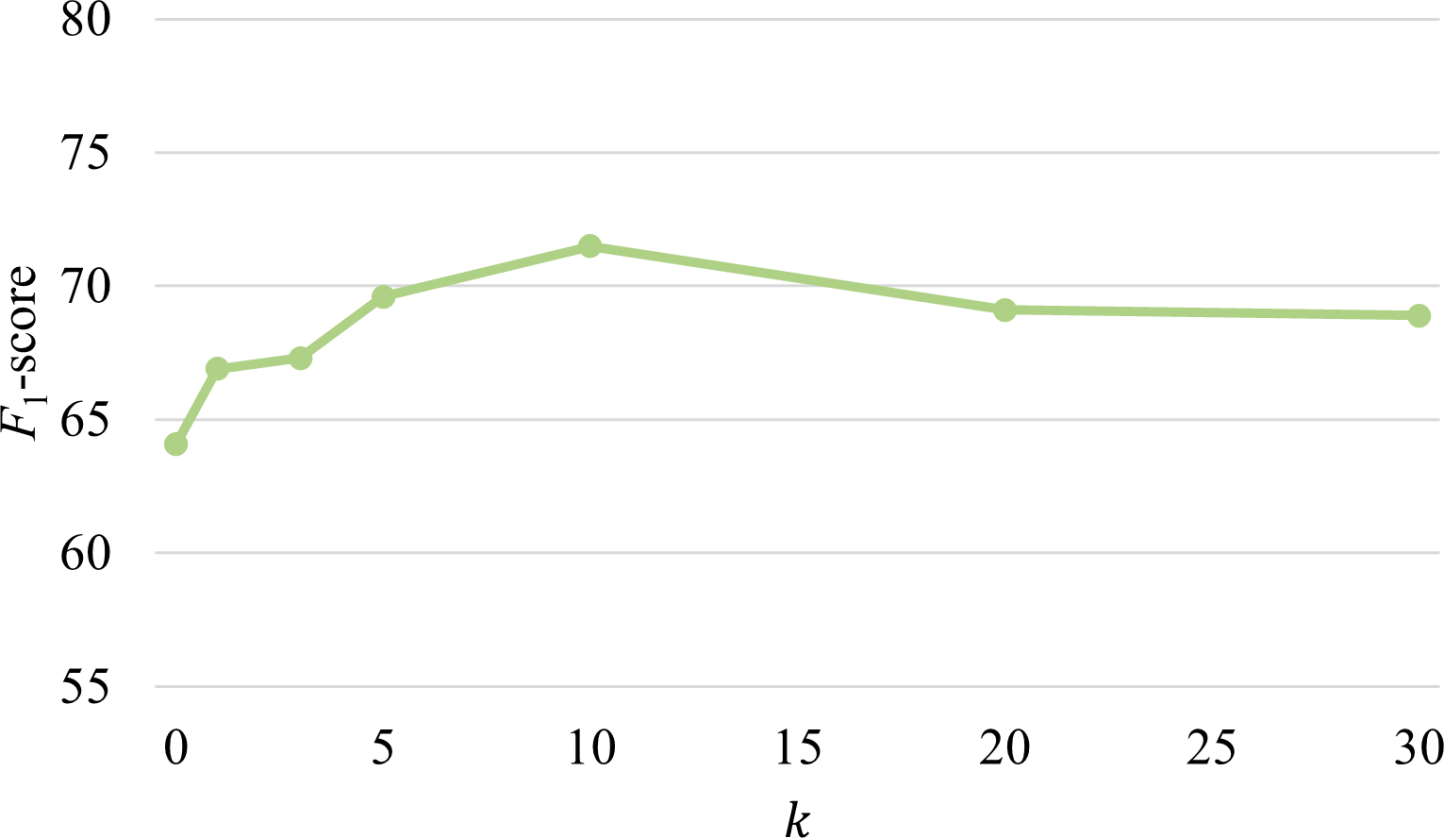
Different knowledge retrieval settings and the corresponding *F*_1_-scores.

We analyzed the impact of parameter k, which controls the number of retrieved reasoning paths in the RAG module. The evaluation across k values [1, 3, 5, 10, 20, and 30] is shown in Figure 3. The *F*_1_-scores indicate that small increases in k initially improved performance, but after reaching an optimal point (k=10), further increases led to gradual accuracy decline. This pattern demonstrates the effectiveness of our reasoning path postprocessing, as it successfully prioritizes the most relevant reasoning paths while filtering out potentially misleading or irrelevant information.

**Figure 4.**
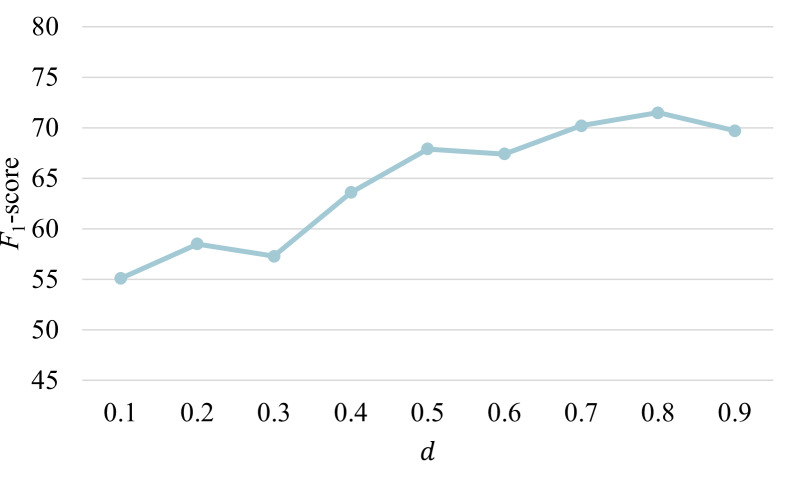
Different damping factor settings and the corresponding *F*_1_-scores.

To study the impact of the damping factor d, where 1-*d* is the probability of randomly jumping to other nodes, we varied d within the range of [0.1, 0.9]. As shown in Figure 4, the performance of Yaoshi-RAG initially improved with increasing d, reached the peak (d=0.8), and subsequently declined. This trend aligns with the commonly adopted value (d=0.85) proposed by Page et al [47] and is consistent with previous findings [48,49]. Specifically, when d is too small, the resulting PageRank distribution is dominated by random jumps, which weakens the influence of the graph structure and yields less meaningful entity rankings. Conversely, when d approaches 1, the distribution becomes nearly uniform, and the convergence of the power iteration method slows significantly, posing computational challenges.

We evaluated the effectiveness of Yaoshi-RAG’s retrieval strategy against multiple baselines. For sparse retrieval, we used BM25 [50] and followed DecAF [51] by transforming 1-hop subgraphs of topic entities into text. In KG embedding–based methods (TransE [52] RAG and RotatE [53] RAG), we ranked triples by embedding similarity. We also extracted the 2-hop subgraph to prioritize semantically relevant triples from query entities’ neighborhood. All baseline methods retained the top 30 triples as retrieval results. Table 4 shows the performance comparison of different retrieval strategies. Yaoshi-RAG achieved superior performance with the highest Hits@1 (84.2%) and *F*_1_-score (71.5%).

**Table 4.** The performance comparison of retrieval strategies.

Retrieval strategies	Hits@1	*F*_1_-score
BM25 (2009)	72.8	65.3
TransE RAG (2013)	82.6	69.9
RotatE RAG (2019)	81.9	68.5
2-Hop Subgraph	75.4	67.2
Yaoshi-RAG (Ours)	*84.2* ^*a*^	*71.5*

a Values in italics indicate the best performance for each metric.

To evaluate the impact of key modules in Yaoshi-RAG, we compared three variants: (1) a variant replacing the UKG with a deterministic KG, in which all triples were assumed to be fully reliable (ie, confidence scores were set to 1); (2) a variant removing the postprocessing of retrieval results, in which all retrieved reasoning paths from the UKG were directly input into the LLM without ranking or filtering; and (3) a variant randomly generating relation paths to guide the retrieval of reasoning paths.

As shown in Table 5, using a deterministic KG leads to a performance decline, highlighting the importance of incorporating uncertainty information derived from the UKG. Performance degrades further when postprocessing is removed, due to the inclusion of noisy and irrelevant reasoning paths. This confirms the effectiveness of leveraging weighted PageRank and triple confidence scores in identifying relevant reasoning paths. The variant using randomly generated relation paths for retrieval performs the worst among all settings, emphasizing the necessity of a retrieval strategy that generates faithful relation paths.

**Table 5.** Ablation studies of Yaoshi-RAG.

Model	Hits@1	*F*_1_-score
Yaoshi-RAG (Ours)	*84.2* ^*a*^	*71.5*
w/o UKG^b^	80.7	67.9
w/o post-processing	75.3	66.2
w/ random relation paths	65.4	54.2

a Values in italics indicate the best performance for each metric.

b UKG: uncertain knowledge graph.

Textbox 5 demonstrates an actual case of a dietary question and the answer generated by our proposed framework. The framework delivers a comprehensive response to a patient presenting peripheral heat sensations and sleep disturbances. By leveraging reasoning paths derived from the MFH KG, the framework establishes clear relations between reported symptoms, underlying constitutional patterns, and appropriate dietary recommendations. The framework also enriches its recommendations by incorporating detailed food entities’ attributes from the KG into the LLM, thereby enhancing the specificity of guidance regarding consumption methods and other relevant details. This case demonstrates how Yaoshi-RAG effectively translates MFH principles into clear and practical guidance, helping patients better understand the recommendations and adhere to the suggested treatments.

Textbox 5. **An actual case of a dietary question and the answer generated by our proposed framework.**

User query:

Lately my hands and feet keep feeling hot, and I’m having trouble sleeping with lots of dreams. Are there any food you’d recommend for this?

我最近手足心热、失眠多梦，请给我推荐一些食材。 Top-3 reasoning paths:

1. (Heat in Hands and Feet, Related Constitution, Yin-deficient Constitution) → (Yin-deficient Constitution, Suitable Food, Lily Bulb)
2. (Heat in Hands and Feet, Related Constitution, Yin-deficient Constitution) → (Yin-deficient Constitution, Suitable Food, White Fungus)
3. (Insomnia and Vivid Dreams, Influencing Factor, Disharmony between Heart and Kidney) → (Disharmony between Heart and Kidney, Suitable Food, Mulberry)

1. (手足心热, 关联体质, 阴虚质) → (阴虚质, 适宜食物, 百合)
2. (手足心热, 关联体质, 阴虚质) → (阴虚质, 适宜食物, 银耳)
3. (失眠多梦, 影响因素, 心肾不交) → (心肾不交, 适宜食物, 桑葚)

Attributes of related food entities:

[Name], [Primary Effects], [Consumption Methods], [Indications], [Contraindications]

Generated answer:

Based on the symptoms you described—Heat in Hands and Feet, along with Insomnia and Vivid Dreams—the following analysis and dietary recommendations are provided according to Traditional Chinese Medicine (TCM) principles:

TCM syndrome differentiation analysisHeat in Hands and Feet is a typical manifestation of “Five-Center Heat” syndrome. When accompanied by Insomnia and Vivid Dreams, the condition may be attributed to three primary constitution types: (1) Yin Deficiency with Excessive Fire: Insufficient Yin fluids in the body lead to internal heat, disturbing the Shen (spirit) and causing insomnia. (2) Disharmony between Heart and Kidney: The inability of Kidney Water to balance Heart Fire results in excessive Heart Fire, affecting sleep quality. (3) Liver Blood Deficiency: A deficiency in Yin and Blood leads to an unsettled spirit, causing frequent dreams and light sleep. These syndromes often intertwine, requiring dietary adjustments to nourish Yin, reduce internal heat, and harmonize the Heart and Kidney.Recommended food and recipes

- Lily, lotus seed, and white fungus soup: Efficacy: Lily calms the mind, Tremella nourishes Yin and moistens dryness, and lotus seed strengthens the kidney and astringes essence. Together, they create a “Metal-Water Mutual Generation” effect. Preparation: Soak 15 grams of Tremella in cold water until fully expanded, then tear it into small pieces. Add 30 grams of fresh lily and 20 grams of decored lotus seeds to 800 mL of water and simmer for 1 hour. Add rock sugar before serving. Best time to consume: Between 3 and 5 PM (Shen Hour), when the bladder meridian is active, enhancing Yin nourishment.
- Mulberry and black sesame paste: Efficacy: Mulberries nourish the liver and kidneys, while black sesame replenishes blood and moisturizes dryness, making them an ideal pairing for dual liver-kidney nourishment. Preparation: Lightly toast 50 grams of black sesame over low heat and then blend it with 20 grams of dried mulberries and 30 grams of glutinous rice with 600 mL of water into a smooth paste. Best time to consume: About 2 hours before bedtime for a calming effect.

Precautions and lifestyle adjustments

- Dietary restrictions: Avoid spicy and heat-inducing food (eg, chili and Sichuan pepper), warming tonics (eg, lamb and longan), and stimulants such as strong tea and coffee.
- Lifestyle recommendations: Prepare for sleep before 9 and 11 PM (Hai Hour). Practice the “Shaking Head and Wagging Tail to Reduce Heart Fire” movement from the Eight-Section Brocade (Ba Duan Jin). Engage in calligraphy or meditation to calm the mind.

The above plan should be followed continuously for at least 2 weeks to see effects. If diarrhea occurs, discontinue the use of Poria. For patients with diabetes, it is recommended to substitute rock sugar with xylitol. If symptoms persist, seek medical attention promptly for tongue and pulse diagnosis.

根据您描述的手足心热、失眠多梦症状，结合中医辨证及食材特性分析如下:

中医辨证分析

手足心热属于“五心烦热”的典型表现，伴随失眠多梦可综合辨证为三类体质问题：其一为阴虚火旺，因体内阴液不足导致虚火内生，上扰心神引发失眠；其二属心肾不交，肾水无法上济心火，致使心火亢盛影响睡眠质量；其三与肝血不足相关，阴血亏虚致使魂不守舍，出现多梦易醒症状。这三种证型常相互交织，需通过滋阴降火、交通心肾的食疗调理。

推荐食材与烹饪方案

- 百合莲子银耳羹：功效配伍：百合清心安神，银耳滋阴润燥，莲子固肾涩精，三者协同形成“金水相生”之效。制作方法：取银耳15克冷水泡发后撕小朵，配鲜百合30克、去芯莲子20克，加水800毫升炖煮1小时，起锅前加冰糖调味。建议申时（下午3-5点）食用，此时膀胱经当令，可增强滋阴效果。
- 桑葚黑芝麻糊：功效配伍：桑葚滋补肝肾之阴，黑芝麻养血润燥，两者搭配实现肝肾同补。制作方法：将文火炒香的50克黑芝麻与20克桑葚干、30克糯米混合，加600毫升水用破壁机打成米糊。适宜睡前2小时温服，有助宁神助眠。

注意事项与生活调摄

- 饮食禁忌：避免辛辣助火之物（如辣椒、花椒）、温热补品（如羊肉、桂圆）及咖啡浓茶等兴奋性饮品。
- 生活建议：保证亥时（21-23点）前准备入睡，日常练习八段锦“摇头摆尾去心火”招式，并通过书法、冥想安神定志。

以上方案需连续食用2周以上见效，若出现腹泻可暂停茯苓，糖尿病患者建议以木糖醇替代冰糖。症状持续者应及时就医进行舌脉诊察。

## Discussion

### Principal Findings

Evaluation results indicate that our framework improved the performance of all evaluated LLMs, showcasing its robust adaptability. High-performing models, such as DeepSeek-R1, exhibited marked improvements when enhanced by the MFH KG. This suggests that the MFH KG offers valuable context about ingredient relations, therapeutic properties, and dietary principles, which might not be fully captured during the models’ pretraining. Remarkably, even LLMs with fewer parameters showed performance improvement when integrated with the MFH KG. These results highlight the effectiveness of the proposed framework, which combines the contextual reasoning capabilities of LLMs with the rich and structured knowledge of KGs, making it a powerful tool for MFH-based dietary recommendations.

The human evaluation results revealed that the KG-augmented LLM demonstrated comprehensive improvements. The augmented LLM exhibited improved rationality through the relations between symptoms and dietary recommendations provided by the MFH KG. It also achieved superior explainability by integrating theoretical reasoning paths. Furthermore, the framework demonstrated increased user acceptability with more clinically applicable suggestions. The augmented LLM also maintained greater consistency across varying inputs. These findings collectively indicate that RAG can effectively enhance LLMs’ capabilities with domain-specific knowledge.

The parameter analysis and ablation studies validate the effectiveness of our postprocessing strategy applied to the retrieved reasoning paths. Retrieving more information does not necessarily lead to performance improvement and may instead introduce irrelevant or noisy information [54], thereby degrading the quality of recommendations. By incorporating triple confidence scores from UKG reasoning, the framework prioritizes informative reasoning paths for dietary recommendations. The performance decline beyond k=10 indicates that confidence-based ranking effectively distinguishes informative reasoning paths from less relevant ones. This underscores the importance of uncertainty modeling in TCM knowledge representation, particularly given the heterogeneous quality of evidence. By measuring confidence scores, the inherent uncertainty in MFH principles can be better managed, prioritizing reliable relations while mitigating the influence of less trustworthy ones.

### Limitations

Several limitations of this study should be acknowledged. The MFH KG was constructed using OpenIE, which may introduce inaccuracies and cannot cover all knowledge on MFH principles. Besides, we did not fine-tune the LLMs specifically for relation path generation, which may have constrained the retrieval effectiveness of our framework, particularly for complex cases involving multiple symptoms or conditions. Addressing these limitations in the future would likely further enhance the LLMs’ clinical uses and recommendation quality.

### Conclusions

This study shows Yaoshi-RAG, a new framework that enhances LLMs’ capabilities in generating MFH dietary recommendations through the integration of a UKG. This framework uses multistep LLM calls for OpenIE to automatically construct an MFH KG, incorporating UKG reasoning to measure the confidence of existing triples and complete missing triples. Upon receiving user queries, the framework implements a reasoning path–based knowledge retrieval strategy, extracting relevant reasoning paths from the MFH KG and optimizing the retrieval results through a postprocessing mechanism. Experimental evaluations demonstrate that DeepSeek-R1 is the best-performing base model for MFH-based dietary recommendation generation. This framework facilitates dietary recommendations that adapt to individual health conditions and symptom requirements while considering diverse ingredients and dishes, accompanied by comprehensive explanations grounded in MFH principles. In the future, we plan to focus on implementing more effective knowledge extraction methodologies, expanding our KG through additional MFH literature, exploring optimized retrieval strategies, and fine-tuning open-source LLMs to further improve the accuracy and reliability of the generated dietary recommendations.

## Supplementary material

10.2196/75279 Multimedia Appendix 1 Prompts for medicine food homology knowledge graph construction.

## Abbreviations

KG: knowledge graph
LLM: large language model
MFH: medicine food homology
OpenIE: open information extraction
RAG: retrieval-augmented generation
TCM: Traditional Chinese Medicine
UKG: uncertain knowledge graph
