# Supplementary material for: Leveraging Retrieval-Augmented Large Language Models for Dietary Recommendations With Traditional Chinese Medicine’s Medicine Food Homology: Algorithm Development and Validation
Source: JMIR Med Inform. 2025 Aug 21;13:e75279. doi: 10.2196/75279 (PMC12370266; doi:10.2196/75279)
Supplement: Multimedia Appendix 1 [file medinform-v13-e75279-s001.docx]

## Appendix

Textbox S1. Prompt for corpus refinement.

| You are required to analyze the given paragraph D, considering its preceding paragraph D-1, following paragraph D+1, and the overall topic of the document. Your task is to determine whether paragraph D contains knowledge related to the concept of "medicine food homology".  Task Instructions:  1. Extract and highlight the core concepts of paragraph D to enhance clarity and coherence.  2. Ensure the optimized paragraph is written in formal academic style, avoiding abrupt sentence breaks.  3.Maintain logical consistency and smooth transitions between D-1, D, and D+1.  4.Remove irrelevant content. In the optimized version, sentences unrelated to TCM "food-medicine homology" should be deleted.  5. Output format: {"optimized_paragraph": "(Please output the optimized paragraph.)"}. The content inside "optimized_paragraph" must be in Chinese.  Paragraph D: {D}  Paragraph D-1: {D-1}  Paragraph D+1: {D+1}  Document Topic: {document_topic}  Please strictly follow the above instructions and return only the required JSON format: |
| --- |

Textbox S2. Prompt for relation extraction.

| You are constructing the ontology for the "medicine food homology" knowledge graph in Traditional Chinese Medicine. Your task is to identify whether the text contains knowledge that requires introducing new relation types beyond those already defined in the ontology.  Task Instructions:  1. If most triplet relations in the text can be expressed using the existing relation types, return "None".  2. If new relation types are needed, output them in a JSON list.  3. The relation types should have clear semantics, capable of encompassing a broad range of triplets, avoiding unnecessary updates.  4. Directly output the new relationship types to be added, without additional text.  Existing Relations: {existing_relations}  Text: {text}  Please strictly follow the above instructions and return only the required JSON format: |
| --- |

Textbox S3. Prompt for relation validation.

| You are constructing the ontology for the "medicine food homology" knowledge graph in Traditional Chinese Medicine. Your task is to determine whether the following relation type is suitable for inclusion in the ontology.  Task Instructions:  1. The relation should be clearly distinct and should not overlap with or be too similar to existing ones.  2. The relation should provide new, valid semantic information, avoiding redundancy.  3. Relationships that do not meet these criteria should not be added.  4. If a relation can be added but needs modification, please return the optimized version.  Existing Relations: {*existing_relations*}  Relation to Evaluate: {*relation*}  Please output either "None" or the optimized relationship type directly, without any additional text: |
| --- |

Textbox S4. Prompt for triple extraction.

| Please perform Open Information Extraction on the following text and extract the entity-relation triples related to "Medicine Food Homology" in Traditional Chinese Medicine, based on the provided relation types.  Task Instructions:  1. Only extract triplets that match the existing relation types.  2. Explicitly annotate food entities with [Food] and dish entities with [Dish].  3. Strictly adhere to the output format: (Entity1 [Tag], Relation, Entity2 [Tag])@(Entity1 [Tag], Relation, Entity2 [Tag]), where [Tag] is [Food], [Dish], or [Null]. Each triplet should be connected by "@".  4. Do not include any extra text or explanations.  Relation Types: {*existing_relations*}  Text to Process: {*text*}  Please strictly follow the above instructions and return only the required format: |
| --- |

Textbox S5. Prompt for updating attributes.

| Given the current attributes of the food entities, reference the source passages to update and enrich the following attributes.  Task Instructions:  1. Update the attributes listed above with the most relevant and accurate information from the source passages.  2. Output the updated information in the following JSON format: {"food_entity": {"name": "{updated_name}", [Other attributes…]}}. The attributes must be written in Chinese.  Attributes: {*attributes*}  Text to Process: {*text*}  Please strictly follow the above instructions and return only the required JSON format: |
| --- |
